# An autoencoder learning method for predicting breast cancer subtypes

**DOI:** 10.1371/journal.pone.0327773

**Published:** 2025-07-23

**Authors:** Zahra Rostami, Kavitha Mukund, Maryam Masnadi-Shirazi, Shankar Subramaniam

**Affiliations:** 1 Department of Computer Science and Engineering, University of California San Diego, San Diego, California, United States of America; 2 Department of Bioengineering, University of California San Diego, San Diego, California, United States of America; 3 Amazon, Seattle, Washington, United States of America; 4 Department of Cellular and Molecular Medicine, University of California San Diego, San Diego, California, United States of America; The University of Queensland Faculty of Medicine, AUSTRALIA

## Abstract

Heterogeneity of breast cancer poses several challenges for detection and treatment. With next-generation sequencing, we can now map the transcriptional profile of each patient’s breast tissue, which has the potential for identifying and characterizing cancer subtypes. However, the large dimensionality of this transcriptomic data and the heterogeneity between the molecular profiles of breast cancers poses a barrier to identifying minimal markers and mechanistic consequences. In this study, we develop an autoencoder to identify a reduced set of gene markers that characterize the four major breast cancer subtypes with the accuracy of 82.38%. The reduced feature space created by our model captures the functional characteristics of each breast cancer subtype highlighting mechanisms that are unique to each subtype as well as those that are shared. Our high prediction accuracy shows that our markers can be valuable for breast cancer subtype detection and have the potential to provide insights into mechanisms associated with each subtype.

## Introduction

Breast cancer (BC) is a highly heterogeneous disease that displays striking molecular and clinical diversity between its subtypes. In this paper, we define the four subtypes based on hormone receptor (HR) status (ER, PR, HER2), namely, luminal A (ER + , PR + , HER2-), luminal B (ER + , some PR + , and HER2- or HER2+), the basal-like subtype as triple-negative, TNBC (ER-, PR-, HER2-), and HER2-enriched (ER-, HER2+). Each subtype has both shared and distinct mechanisms underlying its progression and requires distinct therapy. Luminal B, HER2-enriched, and TNBC have poorer clinical outcomes compared to luminal A. With the advent of transcriptomic methods, there is an increasing interest in identifying unique marker panels, from blood and tissue that can establish cancer subtypes leading to treatment recommendations. A major challenge in analyzing transcriptomic data lies in effective contextual interpretation [1] given that the transcriptional changes are measured and captured over thousands of genes, across cancer subtypes. This has created a need for researchers to develop new strategies for dimensionality reduction which involves mapping high-dimensional gene expression data to lower dimensional space, i.e., reducing the number of genes while retaining the important and useful properties of the original data.

In cancer genomics, machine learning methods have played an important role in dimensionality reduction of transcriptomic data, to identify potential markers that allow for cancer detection and subtyping [2]. For example, a support vector machine (SVM) model has been used on spatial transcriptomic data from breast tissue sections [3], to detect expression signatures of expert annotated regions of non-malignant, ductal carcinoma in situ, and invasive ductal carcinoma. Similarly, a recent work [4] has employed a convolutional neural network model to characterize BC using spatial transcriptome profiles and immunohistochemistry images of BC tissue sections. A point-wise linear model, composed of a deep neural network block and a logistic regression block, has been developed to predict subtypes of breast cancer [5]. In another study [6], two machine learning algorithms have been used to discriminate between BC and normal cells and perform BC subtyping. Their learning pipeline involved principal component analysis (PCA) followed by discriminant function analysis (DFA) in one pipeline (PCA-DFA) and by SVM in the other (PCA-SVM).

In this study, we design an autoencoder to address transcriptomics data complexity and identify a reduced set of markers characterizing the distinct subtypes of BC. Autoencoders are a type of artificial neural network whose architecture deliberately constrains the network, allowing for it to learn efficient ways of representing the input data [7]. Autoencoders also offer the ability of choosing the size of the latent representation [8]. They have been applied to various tasks including dimensionality reduction [9,10], information retrieval [11], facial recognition [12], anomaly detection [13], and biological data interpretation. For example, an autoencoder with 30-dimensional latent space has been used [14] to characterize cell types from gene expression data, by connecting the latent features to different biological processes and pathways in the cell. Vigil et al. [15] have employed a convolutional deep autoencoder to detect breast lesions and extract radiomic biomarkers using ultrasound images. Wang et al. [16] have used an autoencoder for dimensionality reduction and visualization of single-cell RNA-seq (scRNA-seq) and have argued that their method has broader compatibility compared to other methods. In another study [17], an autoencoder has been used to deconvolute biologically meaningful modules encoded in the middle layer of the network, from scRNA-seq measurements. Wang et al. [18] have performed high-resolution cell subtyping using a deep autoencoder and have shown that their model outperforms existing methods for batch effect removal.

Autoencoders use a nonlinear transformation to project data into a lower-dimensional space, where the original features are no longer explicitly preserved. Comparable deep learning studies of BC transcriptomic data often try to partition the reduced subspace into multiple groups where each subtype is more pronounced in a particular group. However, these approaches usually fail to establish a clear relationship between the identified latent features and the original features in the data, and thus, cannot map the resulting partitions in the compressed representation back to the original feature space. To address this limitation, we integrated feature selection into the transition from high-dimensional to low-dimensional space. This approach allowed us to track the original features that most strongly influence the compressed representation of each subtype, thereby identifying genes potentially contributing to specific BC subtypes.

Our method effectively addresses the complexity of transcriptomic data by leveraging the nonlinearity incorporated in the autoencoder model. Transcriptomic data is inherently high-dimensional and often exhibits nonlinear relationships among genes due to complex biological interactions driven by regulatory networks and cooperative biological pathways. Traditional linear dimensionality reduction methods, such as PCA, may fail to capture these intricate relationships. In contrast, the autoencoder uses non-linear activation functions within its neural network architecture, enabling it to model and learn from these non-linear interactions effectively. In the context of breast cancer, this allows the autoencoder to detect subtle patterns in gene expression data that might otherwise remain hidden in a linear framework. Our study focuses on identifying subtype-specific marker genes that capture the unique biological characteristics of each breast cancer subtype. To achieve this, we trained the autoencoder separately for each subtype, and we systematically evaluate the ability of the low-dimensional feature representation derived from our model to characterize BC subtypes via two approaches. First, we perform multi-class classification of the subtypes via a Random Forest (RF) [19] algorithm using the features identified by the model (see Methods). Second, we characterize the profile of the discriminatory feature/gene sets identified in each breast cancer subtype using functional analyses. Our multi-class classification using the RF algorithm resulted in the accuracy of 82.38%, and our functional analyses highlight the subtype-specific relevance of our gene sets.

## Materials and methods

### Data collection and preprocessing

We define the four subtypes of BC based on the status of hormone receptors, HR (ER, PR, HER2), namely, luminal A (ER+, PR+, HER2-), luminal B (ER+, some PR+, and HER2- or HER2+), the basal-like subtype as triple-Negative, TNBC (ER-, PR-, HER2-), and HER2-enriched (ER-, HER2+). Using TCGAbiolinks [20], we collected gene expression data of 1215 primary cancers from GDC harmonized data covering four different subtypes of BC, i.e., triple-negative (TNBC), HER2-enriched, luminal A and luminal B (https://www.cancer.gov/tcga). We used the HTSeq-FPKM data type. The downloaded data primarily contained 56,424 genes. We removed the genes that had expression value of zero across more than 75% of the samples resulting in a total of 36,377 genes. We also retrieved the clinical information in the GDC database from the BCR Biotab (tsv files parsed from the original source of the data) using TCGAbiolinks. Based on the status of hormone (estrogen and progesterone) receptor and HER2 available in the clinical information, the gene expression data was grouped into four classes of BC subtypes. This process resulted in 148, 55, 589, and 170 samples for TNBC, HER2-enriched, luminal A, and luminal B cancers, respectively. We then detected the outliers by calculating the mean and standard deviation of the genes and removed those that were more than one standard deviation away from the mean. As a result, the final number of genes was 28,988 which was used for our downstream analysis.

### Autoencoder

**Fig 1:**
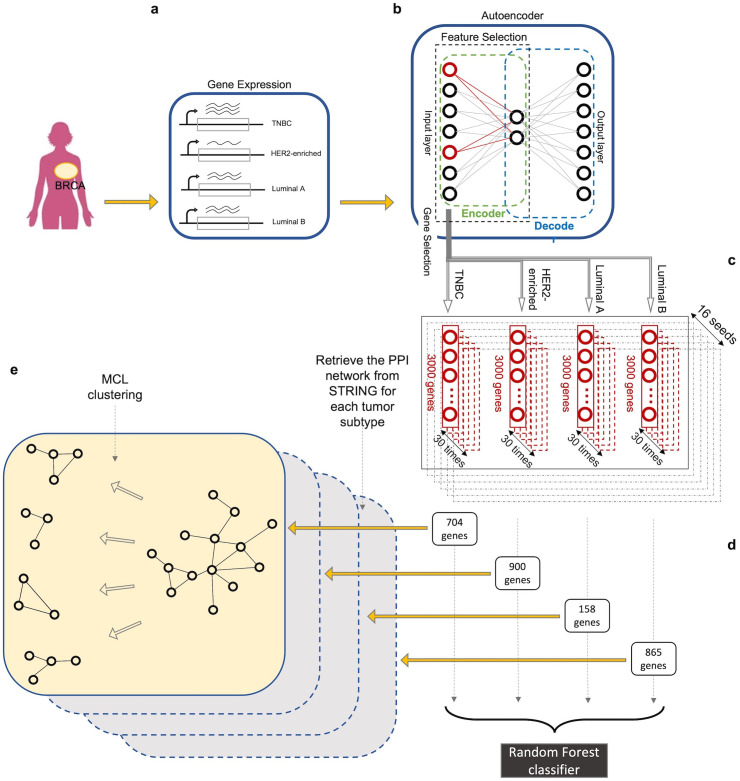
Schematic of our autoencoder-based feature extraction framework. a) We collect data and categorize it into the four subtypes of breast cancer, namely TNBC, HER2-enriched, luminal A, and luminal B. The gene expression data for each subtype is passed to the autoencoder one at a time. b) For each subtype, we perform autoencoder-based feature inference and select the top 3000 genes that have the highest feature scores according to our algorithm. c) For each subtype, we run the autoencoder 30 times with a fixed seed and find the overlap of the resulting gene sets (each containing 3000 genes). We repeat this whole step with 16 different random seeds. d) Taking the overlap of the results from all 16 seeds for each subtype, we identify 704, 900, 158, and 865 marker genes for TNBC, HER2-enriched, luminal A and luminal B cancers, respectively. e) The marker genes are examined via two routes: first, multiclass classification using RF; second, assessment of the protein-protein interactions of the marker gene for each subtype separately. The latter involves MCL clustering of the PPI network derived from the marker genes of each cancer to capture distinct functional modules associated with each BC subtype.

Autoencoder is an artificial neural network that can learn efficient latent representations of the data with no supervision. It consists of an input layer, output layer (whose size is equal to the input layer) and one or multiple layers in between. The smallest layer lies in the middle of the network (see Fig 1b). Essentially, an autoencoder tries to learn a transformation matrix that reconstructs the input in the output layer. This reconstruction is performed via two sequential phases: encoding and decoding. The input first gets encoded in the middle layer and then, it is decoded in the output using the *codings* of the middle layer. Since the number of nodes in the middle layer is smaller than those in the input (and the output) layer, when the network is trained properly, the activation of the middle layer can be treated as a compressed (latent) representation of the input. We used this property and traced back the features that contribute the most to creation of the compressed representation. In each class of cancer subtype, these features (or genes) serve as the most important discriminants, i.e., markers of that particular class. To identify the features, we employed a sparse-learning-based technique. In other words, we made the weight matrix that maps the input to the middle layer, sparse (i.e., some weights are close or equal to zero) by adding a regularizing term to the objective function (details explained in the next part). In machine learning, regularization is often used to prevent overfitting, which ensures that the model does not lose its ability to generalize well to new unseen data. In this work, however, not only the regularizing term was used to force the model to have weights no greater than needed to perform well on the training data [21], but also it served as the basis for our feature selection. The bigger a weight the more significant is the corresponding feature (gene) in distinguishing the cancer subtype.

Our autoencoder was composed of three fully connected feed-forward layers with 28988, 500, and 28988 nodes, respectively. We defined the encoder and the decoder functions as $f(X)=\sigma_{1}(XW^{\left(1\right)})$ and $\hat{X}=g\left(f(X)\right)=\sigma_{2}(f(X)W^{\left(2\right)})$, respectively; where X is the input, and $\hat{X}$ is the network’s output. $\sigma_{1}$ and $\sigma_{2}$ are the activation function of the hidden layer (or the middle layer) and the output layer, respectively. Here, we used rectified linear units (ReLU) activation function for both these layers. $W^{\left(1\right)}$ and $W^{\left(2\right)}$ are the weight matrices of the first and the second layer, respectively. ${Wij}^{\left(l\right)}\;$ denotes the weight (learnable parameter) connecting i-th neuron in layer (l−1) to j-th neuron in layer l.

### Row-sparsity regularization and feature selection

Let $X={\left[x_{1},{\;x}_{2},\;\ldots ,\;x_{n}\right]}^{T}\in R^{n\times d}$ where n is the number of samples, and d is the dimension of samples (i.e., the number of features or genes per sample). We took h (h<d) as the size of the middle layer (which determines the reduced dimension of the feature space). The weight matrix connecting the input layer to the middle layer is $W^{\left(1\right)}={\left[w_{1},\;w_{2},\;\ldots ,\;w_{d}\right]}^{T}\in R^{d\times h}$ where $w_{i}$ represents the i-th row of W and corresponds to the j-th column of the matrix X (j-th feature). The norm of each row of W indicates the importance of the corresponding feature. If ${\left\|w_{i}\right\|}_{2}$ is close to zero, the corresponding feature (the j-th column of X) makes a little contribution to the latent representation of the data in the middle layer. Conversely, if the j-th feature plays a major role in the latent representation, then the corresponding ${\left\|w_{i}\right\|}_{2}$ must be large. We computed the regularizing term as follows:


$${\left\|W^{\left(1\right)}\right\|}_{2,1}=\sum_{i}^{d}\sqrt{\sum_{j}^{h}{\left(w_{ij}^{\left(1\right)}\right)}^{2}\;}\;.$$ (1)

The regularizing term above was added to the objective function when training the network. In addition, each feature was assigned a score which reflects the importance of that feature. These feature scores were calculated as follows:


$$s_{i}=\sum_{j}^{h}{\left(W_{ij}^{\left(1\right)}\right)}^{2}\;$$ (2)

where $s_{i}$ is the score associated with $w_{i}$ and corresponds to j-th feature in the input data (X). We then sorted the features based on their score value in descending order and selected the first 3000 features (genes).

### Model training

We randomly split the data into training (80%) and test (20%) sets with stratification, equaling the proportion of class labels in both sets. The input and the output of the autoencoder are the feature matrices $X\in R^{n_{v}\times d}\;$and $\hat{X}\in R^{n_{v}\times d}$, respectively, where $n_{v}$ represents the number of samples in class v. The feature matrix of each class was entered into the network one at a time. For training an autoencoder, the objective function to be minimized is typically defined as follows:


$$J(\Theta )=\;\frac{1}{2n_{v}}{\left\|X-\hat{X}\right\|}_{F}^{2}$$ (3)

where $\Theta ={\{W}^{\left(1\right)},\;W^{\left(2\right)}\}$, and $\|.{\|}_{F}$ is the Frobenius norm. We added the regularizing term introduced above to the objective function. This way, the model was penalized based on the size of the weights and encouraged toward using small weights. The objective function was thus reformulated as follows:


$$J(\Theta )=\;\frac{1}{2n_{v}}{\left\|X-\hat{X}\right\|}_{F}^{2}+\lambda {\left\|W^{\left(1\right)}\right\|}_{2,1}$$ (4)

where λ controls the impact of penalty on the model when training the network. In other words, λ introduces a trade-off between the loss and the regularization term. The value of λ can be between 0 (no penalty) and 1 (full penalty). This parameter can also help to avoid overfitting during the training. We set $\lambda =10^{-5}$ for TNBC, $\lambda =10^{-4}$ for HER2-enriched, and $\lambda =10^{-6}$ for luminal A and luminal B cancers. We considered mini-batch gradient descent with the batch size of 64 and used Keras (https://github.com/keras-team/keras) to compute the gradients and Adam optimizer [22] to train the model.

### Model validation

To validate our model, we first used a RF classifier to classify the BC subtypes using the genes identified by our model. Next, we explored the functional profile of the gene sets associated with each cancer subtype.

### RF multi-class classification

To perform the multi-class classification using RF, first we randomly split the entire data into training (80%) and test (20%) sets with stratification, which was to ensure the proportion of samples in the training and test sets are the same. We used Scikit-Learn [23] to build and train the RF classifier. It was trained with 1000 trees and 80% of the data, i.e., the training set (to grow the trees). The remaining 20%, i.e., the test set, was then used to calculate the classification accuracy. To decide on hyperparameters (which control how the trees are grown), we ran a grid search with ten-fold cross-validation. Additionally, since the number of samples across the classes of cancer subtypes was imbalanced, we used a class weighting technique to modify the decision trees accordingly. This technique involves changing the class weights when calculating the impurity score of a given node of the trees, which is a measure of how mixed the samples of different classes are at that node. We used the Gini index to measure the impurity. In decision trees, the Gini index plays a role analogous to cost function. In other words, the objective is to minimize the Gini index at each node of the tree. The Gini index of each node is computed as follows:


$$G_{u\;}=\;1-\sum_{v=1}^{k}{{\;p}_{u,v}}^{2}$$ (5)

where $G_{u}$ is the Gini index of node u, k is the number of classes (here k=4), and $p_{u,v}$ is the ratio of class v samples among the training samples in the $u^{th}$ node. In a regular setting, where the data is balanced, the weights associated with each class are assumed to be one. Here with the imbalanced data, however, we adjusted the weights inversely proportional to the class frequencies in the input data^15^ (see Eq. 7). This gave higher weights to the minority classes, making misclassification cost of those classes larger. Given this, the Eq. 5 was modified as follows:


$$G_{u\;}=\;1-\sum_{v=1}^{k}{{w_{v}\;p}_{u,v}}^{2}$$ (6)

$$w_{v}=\frac{n}{k\cdot n_{v}}$$ (7)

where n is the total number of samples, and $n_{v}$ is the number of samples in class v.

To validate our proposed approach, we conducted an evaluation of the RF classifier’s performance. This assessment encompassed key metrics commonly employed for machine learning model validation, including accuracy, precision, recall, and the F1 score. Furthermore, we computed subtype-specific receiver operating characteristic (ROC) curves and the area under the curve (AUC) values, both individually and in terms of their overall average, to comprehensively gauge the efficacy of our method. These evaluation results are documented in the Results section of this study.

### Functional annotation and module detection

We used clusterProfiler [24] package to perform enrichment analysis of the gene sets obtained from the model for the four cancer subtypes based on biological process, cellular component, and molecular function gene ontologies (GO). ”Simplify” function from clusterProfiler was used to remove redundant GO terms from the output of the enrichment analysis (adj p-value 0.05). of 0.7. cancer

Next, for each cancer subtype, we created a network in Cytoscape that included the genes we identified for that cancer by loading the protein-protein interaction (PPI) data from STRING [25]. We retained only the interactions with a confidence score higher than 0.7. To explore functional profile of each cancer at a more granular level, we performed topological clustering using Markov Cluster (MCL) algorithm [26] to partition the network into different modules based on the interactions. The inflation value of MCL was set at 2.5. Finally, we retrieved the functional enrichment of each module using the stringApp in Cytoscape with an fdr adj p-value <0.05.

## Results

### Categorizing breast cancer data

After performing the preprocessing steps described in the Materials and Methods section, we obtained a final gene expression matrix containing 28,989 features for each subtype and 148, 55, 589, and 170 samples for TNBC, HER2-enriched, luminal A, and luminal B, respectively. This reduced set of genes was subsequently used for all downstream analyses.

### Feature inference using autoencoder

**Fig 2:**
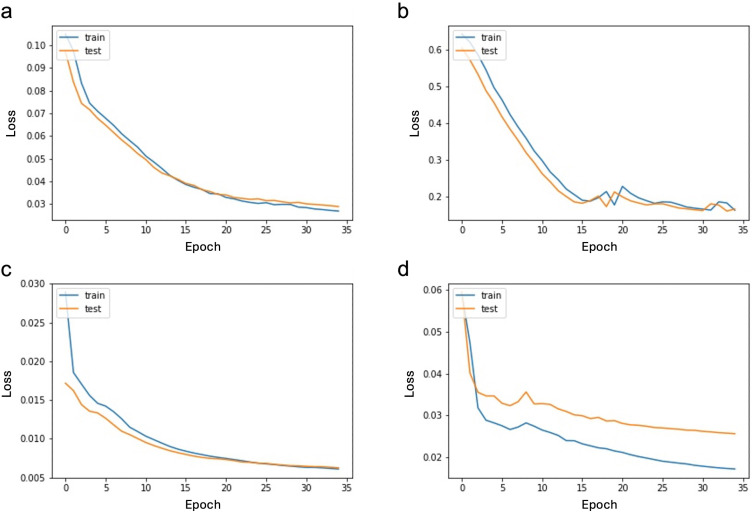
The loss curve representing the performance of the autoencoder for each BC subtype. a) TNBC cancer, b) HER2-enriched cancer c) luminal A cancer, d) luminal B cancer. The x-axis shows the number of epochs. The curves demonstrate that the model fits well on the data, does not overfit, and stays consistent throughout training.

We designed an autoencoder to identify features that are unique to each subtype of BC. Autoencoder is an artificial neural network that usually has a symmetric architecture around its middle layer. A schematic of the autoencoder is shown in Fig 1. The autoencoder tries to find a set of weights that best reconstruct the input in the output layer. The middle layer has the smallest number of nodes compared to other layers and can infer latent variables from the empirical measurements. Using this concept, we trained the autoencoder on each class of cancer subtype separately, to reconstruct the samples of a particular class and get a compressed representation of that in the middle layer of the autoencoder. So, in each run, the complete list of genes obtained in the previous section was entered into the autoencoder and a reduced set of genes that still can reconstruct the input in the output layer with an acceptable error was extracted. We calculated this error by measuring the distance between the reconstruction and the input, using the Mean Squared Error (MSE) loss function. Prior to passing the instances to the autoencoder, we normalized the expression values in the range of [0, 1] using the MinMaxScaler. To ensure effective reconstruction by the model and its ability to generalize to new samples, we utilized 80% of the data for training, while the other 20% served for testing. We used the Adam [22] version of stochastic gradient descent to fit the model. Fig 2 demonstrates model performance for each class through the learning curves over 35 epochs, and highlights that our model achieves a good fit in reconstructing the input, holds steady throughout training, and is not overfitting.

When the autoencoder was successfully trained on the instances of each class (i.e., the model was fit), a dense representation of the data was stored in the middle layer (also known as the bottleneck layer). The encoder model was saved and used for downstream feature extraction. Additionally, to ensure that the best version of our model was used during the whole learning process, we monitored the validation loss at every epoch and saved the weights of the latest best model. In other words, the encoder model that was to be used for feature selection got updated at each epoch only if the new version of the model performed better than the previous one. After all the training data was mapped to a lower dimensional space, we performed feature selection on the new subspace using the best version of the encoder model we had obtained in the previous step. For feature selection, we employed row-sparsity regularization (described in the Methods section) on the weights connecting the input to the middle layer. The features were sorted by feature scores and the top 3000 features with the highest scores were chosen (see Methods for details). We also noted that neural networks are inherently stochastic, and randomness is an important and inevitable part of their configuration, learning and optimization. The most common source of randomness is random initialization of the weights of the network. To account for this stochasticity, we used a fixed random seed for the random number generator and ran the autoencoder 30 times, each resulting in 3000 features. We then found the overlap of all the 30 feature sets. To rule out the potential influence of the random seed and examine the robustness of our algorithm against the seed, we repeated these steps performing 16 independent runs with 16 different random seeds and identified their overlap. This final overlap yielded 898, 612, 247, and 745 marker genes for TNBC, HER2-enriched, luminal A, and luminal B cancers, respectively (Table 1, list of genes is available from authors upon request). The distribution plot of our gene expression data using our identified marker sets associated with each BC subtype is presented in S1 Fig. A workflow diagram providing an overview of our procedure for feature extraction and for validation of our model (explained in follows) is shown in Fig 1.

**Table 1:** The number of samples and the number of marker genes (obtained from the autoencoder) for each BC subtype.

Breast Cancer Subtypes	Number of Samples	Number of Marker Genes
Triple-Negative (TNBC)	148	898
HER2-enriched	55	612
Luminal A	589	247
Luminal B	170	745

### Defining the class-specific feature space

To verify our model-derived features, we first wanted to examine if we can correctly classify our cancer samples with the newly defined, reduced feature space identified in the previous section, using an independent classifier. We used an RF algorithm towards this aim. RF is an ensemble of Decision Trees [27] and has several properties making it appropriate for classification. First, it combines the idea of adaptive nearest neighbor with the bagging method and offers a highly data adaptive inference. Second, it has a greedy nature, and in each step, it introduces extra randomness by searching for the best feature among a random subset of features. By repeating various forms of decision trees, it imposes regularization for effective analysis in problems where the number of features is much larger than the number of samples (which is usually the case in omics data). However, we also explored SVM and Logistic Regression and we find them comparable in performance to the RF method used in this work (see Supporting Information, S1 Table).

**Fig 3:**
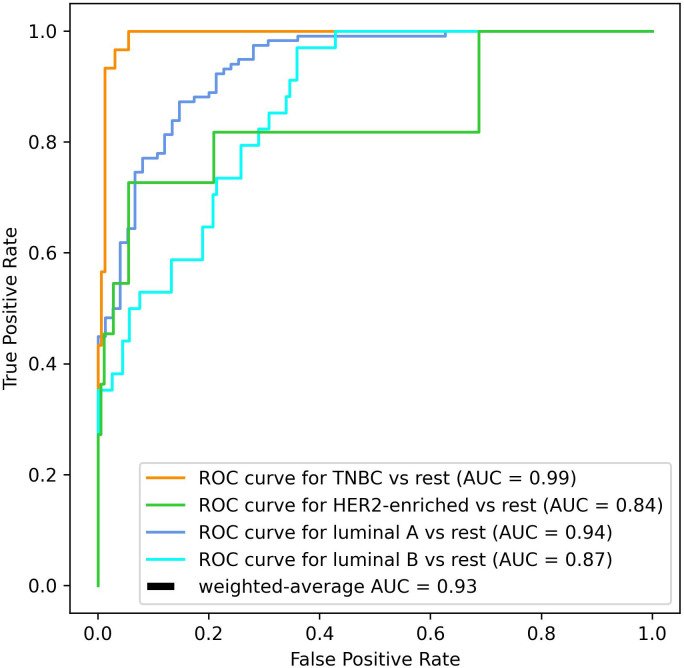
Receiver operating characteristic curves showing the performance of the model.

Since the data downloaded from TCGA was imbalanced (see Table 1), when classifying the cancers, our procedure involved assigning weights to each class such that their magnitude was inversely proportional to class frequency in the input data (see the model validation in the Methods section) [28]. After these adjustments, we were able to classify the data with 82.38% accuracy using the RF algorithm. Also, the weighted-average precision, recall, and F1 score over the four classes reached 83.6%, 82.4%, and 79%, respectively. We further evaluated the overall classification power of the method using the ROC curves and AUC values. Fig 3 demonstrates the ROC curves and AUC when using one-vs-rest multiclass classification strategy. We obtained AUC of 0.99, 0.84, 0.94, 0.87 for TNBC, HER2-enriched, luminal A and luminal B breast cancers, respectively, and the weighted-average ROC curve of all four classes resulted in AUC of 0.93. The weighted-average strategy was used to calculate the average values due to the imbalance of the four classes.

To assess our model’s applicability to diverse populations and clinical settings, we evaluated our model on an independent cohort from the Gene Expression Omnibus (GEO) database [29] under GEO accession GSE81538 [30]. The data contained FPKM counts aligned to GRCh37/hg19 and underwent the same quality control as done in our TCGA set [31]. It included 57, 65, 156, and 105 samples for TNBC, HER2-enriched, luminal A, and luminal B BC, respectively. The multiclass classification of BC subtypes on this new dataset using our identified marker genes resulted in an accuracy of 79.22%. The corresponding ROC curves for this external validation are provided in S3 Fig.

**Fig 4:**
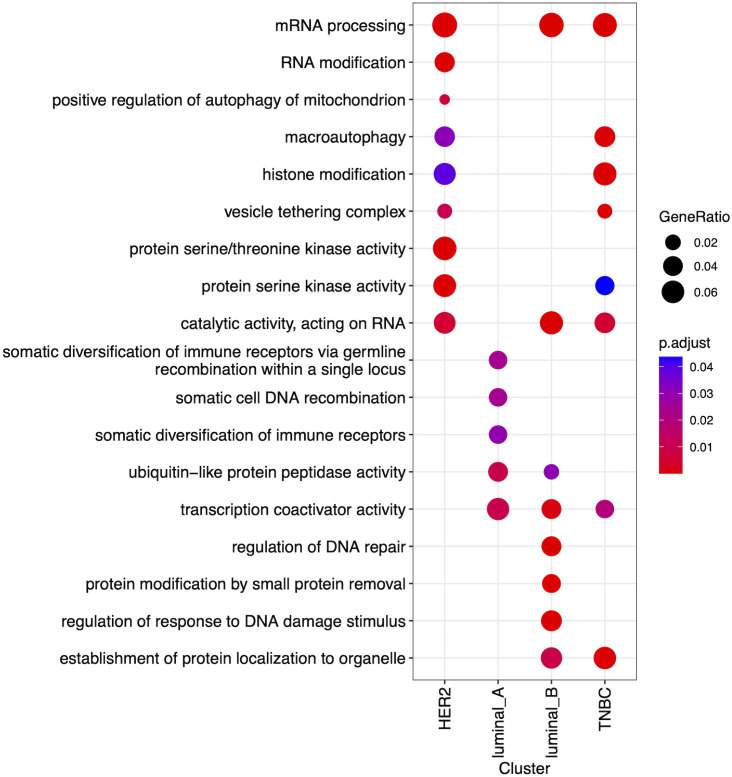
Comparing functional profile of the four BC subtypes using the genes derived from our model. The plot shows the 18 most significant gene ontologies. The node size represents k/n ratio, where n is the size of the list of genes corresponding to a subtype (this number is shown in parenthesis on the x-axis) and k is the number of genes within that list that are annotated to the node. The dot colors indicate the adjusted p-values.

To get broad functional insights from model-derived features, we performed over representation analysis of the gene sets identified for each breast subtype, using biological processes, cellular components, and molecular functions in the GO database via clusterProfiler [24]. The results are shown in Fig 4 which represents 18 most significant biological processes annotated based on the markers of the four subtypes. In order to parse the functional categories into mechanistic modules, we used the subtype-specific features using protein interaction networks to construct mechanistic modules.

To obtain functional modules attributable to subclasses, we extracted protein-protein interactions (via StringDB in Cytoscape) of our model-derived features for each cancer type and clustered them (via MCL clustering, see Methods) to capture the distinct functional and molecular mechanisms. In total, we extracted 243 modules containing more than two genes, with the largest having 98 genes and the smallest with 3 genes. A majority of our modules have been identified in prior scientific literature and our novel modules provide hypotheses for testing in prospective cohorts. Moreover, where the modules presented overlapping functional profiles, we merged them to form super-modules characterizing shared functions between the four cancer subtypes. In the following subsections, we present an analysis of these modules that are either unique or shared between the subtypes.

### Autophagy super-module

**Fig 5:**
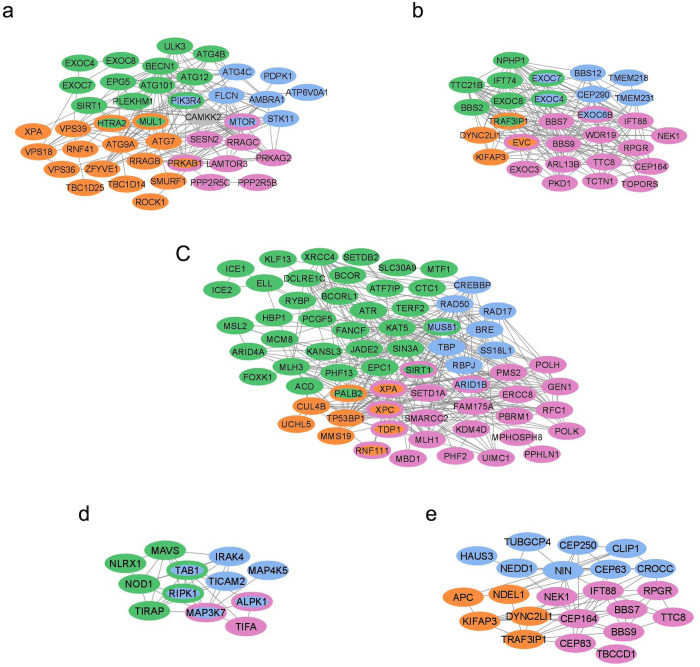
Functional modules and super-modules associated with different BC subtypes. The genes associated with TNBC, HER2-enriched, luminal A and luminal B cancers are shown in green, blue, orange, and pink, respectively; a) Autophagy super-module (CAMKK2, shown in gray, is common between TNBC, HER2-enriched and luminal B cancers), b) Ciliary trafficking machinery and cilium assembly super-module, c) Chromatin organization and remodeling super-module, d) NF-kappa B signaling module, e) Centrosome localization and biogenesis module.

Our findings suggested that all subtypes show autophagy as an endpoint phenotype. Fig 5a shows the genes that are involved in autophagy for each of the subtypes. As shown in Fig 5a, MTOR, PIK3R4, and several autophagy related (ATG) genes (ATG101, ATG12, ATG4B, ATG4C, ATG7, ATG9A) as well as exocyst complex components (EXOC4, EXOC7, EXOC8) were among the genes of the autophagy module. We found MTOR and CAMKK2 as a feature important to both Luminal B and HER2-enriched cancers, while PIK3R4, a regulatory subunit of PI3K complex, was observed to be significant to TNBC and HER2-enriched cancers within the autophagy module.

### Ciliary trafficking machinery and cilium assembly super-module

We identified modules related to ciliary trafficking machinery and cilium assembly in all BC subtypes (Fig 5b). These modules contained several cancer regulators such as Intraflagellar Transport (IFT) complex proteins (IFT74, IFT88), BBSome complex proteins (BBS2, BBS7, BBS9, BBS12), exocyst complex components (EXOC3, EXOC4, EXOC6B, EXOC7, and EXOC8), TTC8, TTC21B, EVC, and NEK1 [32].

### Chromatin organization and remodeling super-module

We retrieved functional modules enriched in chromatin organization and remodeling (Fig 5c). These modules involving heterochromatin assembly, zinc transporters and zinc-binding proteins, and DNA damage repair, were shared between all the subtypes. However, within these modules, distinctive biological processes were associated with specific subtypes. We found telomere maintaining genes (TERF2, CTC1, and ACD), genes involved in zinc homeostasis (MTF1 and SLC30A9), transcription elongation factor complex components (ICE1, ICE2, ELL), and BCL6 corepressors (BCOR, BCORL1) uniquely invoked in TNBC cancers. ATF7IP, an important modulator of chromatin formation involved in heterochromatin assembly was also observed only among our TNBC markers. On the other hand, we found nucleotide excision repair pathway uniquely in luminal A (enriched by CUL4B, XPA, XPC, MMS19, RNF111), and luminal B (enriched by XPA, RFC1, ERCC8, XPC, RNF111, SIRT1, POLK).

### NF-kappa B signaling module

We identified several genes associated with NF-kappa B signaling within our reduced feature space for TNBC and HER2+ (HER2-enriched and luminal B) cancers (see Fig 5d). In this module, a positive regulation of NF-kappaB transcription factor activity and I-kappaB kinase (IKK)/NF-kappaB signaling was characterized by a submodule involving MAVS, NOD1, RIPK1, TAB1, and TIRAP from TNBC cancers, ALPK1, IRAK4, MAP3K7, RIPK1, TAB1, TICAM2 from HER2-enriched cancers, and ALPK1, MAP3K7, and TIFA from luminal B cancers. Additionally, a negative regulation of IKK/NF-kappaB signaling was enriched only in TNBC by two genes: NLRX1, a unique pattern recognition receptor (PRR) and a member of NOD-like receptor (NLR) family; and RIPK1, a member of receptor interacting protein (RIP) family of serine/threonine protein kinases. We note that in TNBC, a higher proportion of genes belonged to upregulation of IKK/NF-kappaB signaling cascade (with FDR = 1.3e-4) rather than to its downregulation (with FDR = 0.014). Other interrelated mechanisms within this module were c-Jun N-terminal kinase (JNK) cascade shared between TNBC and HER2-enriched cancers, and TRIF-dependent toll-like receptor signaling pathway unique to HER2-enriched cancers.

### Centrosome localization and biogenesis module

Our analysis identified an enrichment of centrosome localization and biogenesis in HER2-enriched, luminal A, and B subtypes. As shown in Fig 5e, within this module, markers of luminal A were KIFAP3, DYNC2LI1, TRAF3IP1, APC, and NDEL1; while those from luminal B were IFT88, NEK1, TBCCD1, RPGR, CEP83, CEP164, TTC8, BBS7 and BBS9. Finally, HER2-enriched markers involved in this module were identified as NEDD1, HAUS3, CEP63, CEP250, NIN, CROCC, TUBGCP4, and CLIP1, which play a role in mitotic cell cycle.

### BC subtype-specific drug targets in our reduced feature space

We further explored the potential clinical applications of our identified markers by mining the druggable genome using the Drug Gene Interaction database (DGIdb) [33]. Our analysis revealed several drug-gene interactions involving approved drugs according to DGIdb, suggesting potential avenues for therapeutic intervention. S2 Table represents the top four drug-gene pairs per BC subtype that we identified.

## Discussion

Identification of features characterizing BC subtypes in women provides opportunities for timely diagnoses as well as intervention. Prior research has extensively probed the transcriptional landscape from breast biopsies and have offered insights into luminal, HER2+ and basal cancer subtypes. A significant barrier in deciphering markers and mechanisms from transcriptomic landscapes has been the large dimensionality of data and the significant overlap between the basic mechanisms of cancer development and progression. Here, we introduced an autoencoder method (see Methods) to identify features related to four BC subtypes, namely, luminal A, luminal B, Her2+ and TNBC, using a large patient cohort. The feature space we identified for each subtype presents potential biomarkers that can provide crucial insights into mechanisms unique to each cancer subtype.

In autoencoder implementations, the transition from the original data to the lower-dimensional data is completely nonlinear, and the notion of features in the original data are no longer preserved in the new representation. With this in mind, we sought to identify potential markers of BC subtypes by incorporating the feature selection process within the transition from the high-dimensional to the low-dimensional space. Consequently, we kept track of the original features that best guide the compressed representation of each subtype, and hence, can identify what set of genes may contribute more to a given BC subtype. As presented in the results, our model obtained a validation accuracy and F1 score of 82.38% and 0.79%, respectively, and a weighted-average AUC of 0.93 for classification of the cancer subtypes using the RF classifier trained with our identified features. Despite the simple architecture of our model compared to some state-of-the-art machine learning methods introduced for BC subtyping, it shows comparable or better performance over other methods and further provides mechanistic insights into altered biology in each cancer subtype. For instance, a multi-layer convolutional neural network proposed in [34] shows the accuracy of 77.3% and AUC of 0.83 using the gene expression data to predict BC subtypes. Their base network comprises a convolutional layer followed by a max pooling layer, two fully connected layers, a dropout layer, and another fully connected layer. In another study [35], the authors have developed a deep neural network to identify the four molecular subtypes of BC (luminal A, luminal B, HER2-enriched and TNBC with 277, 40, 11, and 70 samples, respectively) using omics data from TCGA. Their network consists of an encoding subnetwork, which has a fully connected layer with ReLU activation function, and a classification subnetwork, which has a fully connected feed-forward neural network with Softmax activation function. Their model represents an accuracy of 74.3% using the mRNA data, and 78.2% using the multi-omics (mRNA, methylation, and copy number variation) data for multi-class classification of BC. Their reported mean AUC on their independent test dataset is 0.93.

An interesting outcome of our analyses of the markers showed the common functional endpoints across the cancer subtypes albeit through utilization of distinct and alternate molecular cascades. In the case of autophagy (Fig 5a), we observed more metabolic implications in TNBC, which has the lowest survival rate, and HER2+ (luminal B and HER2-enriched) cancers. More specifically, both TNBC and HER2-enriched cancers showed a PIK3R4 (a regulatory subunit of PI3K complex), and the HER2 + cancers an MTOR-associated defects of metabolic changes. However, TNBC markers involved BECN1 as well, which is necessary for the activity of PIK3C3, the catalytic subunit of PI3K complex [36]. Thus, based on our findings and following prior studies [36], in regulation of autophagy, TNBC seemed to involve a specific class of lipid kinases where the PIK3C3/Vps34-BECN1-PIK3R4 complex is formed. This complex produces phosphatidylinositol 3-phosphate (PtdIns3P), which is a key phospholipid required for autophagy initiation and progression [36]. The HER2 + cancers, on the other hand, seemed to involve activation of another class of lipid kinases through MTOR. In particular, the AMPK/MTOR and the canonical AMPK/PI3K/AKT/MTOR cascades were more highlighted in luminal B cancers (by CAMKK2 and MTOR) and in HER2-enriched cancers (by CAMKK2, PIK3R4 and MTOR), respectively. Additionally, FLCN and FNIP1, two regulators of energy homeostasis, were uniquely identified in HER2-enriched cancers. FLCN can form a complex with FNIP and perform as a cancer suppressor via regulation of metabolism through AMPK and MTOR [37]. There is also strong evidence showing the activation of MTOR cascade due to high levels of HER2 [38], which was supported by our findings through several mechanisms and pathways we identified in our HER2 + cancers that target MTOR and change glucose metabolism. Moreover, although luminal A and luminal B shared a lot of similarities in the autophagy machinery, they invoked distinct mechanisms to reach the same endpoint. In luminal B, autophagy seemed to be affected upstream, as seen through MTOR, SESN2, which is a modulator of AMPK/MTOR pathway [39], LAMTOR3, PPP2R5B, and PPP2R5C. PPP2R5B and PPP2R5C encode PP2A protein, which is a direct way for MTOR to act on ATG genes [40]. Therefore, based on the literature, our results suggest that autophagy in luminal B is likely influenced upstream whereas in luminal A, it is affected downstream as it further involves autophagosomal genes (ATG7, ATG9A).

While dysregulation of ciliary machinery was common between the four subtypes, distinct members of the ciliary landscape were detected in the reduced feature space across these subtypes (see Fig 5b). Ciliary dysfunction is associated with a wide spectrum of diseases (ciliopathies) [41]. The loss of primary cilia or inhibition of cilium assembly has been observed to play a role in development of BC [32,42]. Our results showed that all cancers likely exhibit disruption of the cilium assembly. As shown in Fig 5b, the markers included Intraflagellar Transport (IFT) complex proteins (IFT74 and IFT88), BBSome complex proteins (BBS2, BBS7, BBS9, and BBS12), and TTC8, some of which have been observed to be downregulated in BC [32]. However, TNBC, HER2-enriched and luminal B cancers additionally included exocyst-associated markers (EXOC3, EXOC4, EXOC6B, EXOC7, and EXOC8), which suggested that, unlike luminal A, these cancers not only share dysregulation of ciliogenesis but also dysfunction of transport to cilium. Furthermore, EVC was identified at the interface of luminal A and B, the ER+ cancers. This protein is a component of EvC ciliary complex and positively regulates ciliary Hedgehog (Hh) signaling. The type I non-canonical Hh signaling is important in ductal morphogenesis at puberty [43]. It activates proliferative ERα cascade in breast luminal epithelial cells and is downregulated in normal adult breast tissue [44].

Aberrant chromatin organization and remodeling, commonly identified in all the cancers, was orchestrated by distinct mechanisms and pathways across the subtypes (Fig 5c). In TNBC, it involved dysregulation of telomere structure and function, zinc homeostasis and BCL6 corepressor pathways. Telomere dysfunction was seen through TERF2, CTC1 and ACD. Telomere length is strongly associated with aggressiveness of BC [45,46], and reducing the expression of TERF2 has been observed to impair the growth of TNBC cells [47]. The dysregulation of zinc homeostasis in TNBC was through MTF1 and SLC30A9 by utilizing WikiPathway analyses. MTF1 is the major transcription factor involved in intracellular metal homeostasis [48] and a cellular zinc sensor, and SLC30A9 (ZnT9) is an important zinc transporter. Zinc has been shown to play an important role in all hallmarks of successful metastasis, which is the main cause of death in patients with TNBC [49]. Disruption of zinc homeostasis has been reported to increase tumorigenic potential of TNBC cells. Prior studies have also shown the relevance of zinc transporters in tamoxifen resistant BC [49]. BCL6 corepressor pathway, another malfunctioning pathway uniquely invoked in TNBC was seen through BCOR and BCORL1. BCL6 is an important transcriptional regulator in BC and has been reported to be highly expressed in breast cancers. TNBC cells, however, are reported to be more sensitive to inhibition of BCL6 compared to other breast cancer subtypes [50]. A well-characterized class of chromatin alterations in response to DNA damage, nucleotide excision repair, was delineated uniquely at the interface of luminal A and B (ER+), through CUL4B, XPA, XPC, MMS19, and RNF111 in luminal A, and XPA, RFC1, ERCC8, XPC, RNF111, SIRT1, and POLK in luminal B. Mutations in nucleotide excision repair genes are suggested as drivers of ER+ cancers [51].

As shown in Fig 5d, NF-kappa B signaling was characterized by the markers identified for TNBC and HER2+ (HER2-enriched and luminal B) cancers, though via different mechanisms. While HER2-enriched and luminal B cancers demonstrated only the upregulation of NF-kappaB transcription factor activity and I-kappaB kinase (IKK)/NF-kappaB signaling, TNBC showed the downregulation of IKK/NF-kappaB signaling as well. The positive regulation of this signaling cascade in TNBC was achieved through interaction of RIPK1 with MAVS, NOD1, TAB1, and TIRAP, whereas its negative regulation was obtained via interaction of RIPK1 with NLRX1. In the innate immune system, unlike most PRRs that are designed to elicit immune response, NLRX1 is a unique PRR in NLR family that attenuates inflammatory pathways such as NF-kappaB signaling. Recent studies have found that, depending on the context of the cell, NLRX1 can act as a promoter or suppressor of cancerous cells [52,53]. When NLRX1 is expressed in a healthy cell, it can repress cancer-associated characteristics through inhibition of epithelial-mesenchymal transition (EMT) and metastasis. However, the expression of NLRX1 in cancerous cells leads to deregulation of EMT and thus facilitates cancer metastasis and increases the disease burden, especially in more aggressive cancers such as TNBC [52,54].

Dysregulation of centrosome and cell cycles, the hallmarks of cancer, were also identified through our marker genes. We observed enrichment of centrosome localization and biogenesis in the ER+ and HER2 + cancers (Fig 5e). HER2-enriched cancers involved four regulators of PLK1 activity at G2/M transition in the cell cycle, CEP63, CEP250, NEDD1, and HAUS3. PLK1 is the most studied member of Polo-like kinase (PLK) family and plays an important role in mitosis [55]. Studies have shown that PLK1 can modulate development of HER2-driven breast cancers affecting chromosomal instability and cellular growth rate [56]. Additionally, in agreement with our finding, there is compelling evidence that centrosome can directly be regulated by ERα in ER+ cell lines [57].

Our drug-gene analysis identified several clinically actionable interactions, providing new opportunities for personalized treatment strategies for BC subtypes. We found drug-gene pairs such as Docetaxel-XRCC4 for TNBC highlighting potential enhancements to standard chemotherapy regimens. Docetaxel is already a cornerstone in the treatment of metastatic BC [58], and targeting XRCC4 could further refine therapeutic efficacy by addressing DNA damage repair pathways, which are critical in TNBC [59]. For HER2-enriched BC, the identification of Everolimus-FLCN reinforces the therapeutic potential of mTOR pathway inhibition, aligning with previous studies that have demonstrated Everolimus’s effectiveness in HER2-positive BC [60]. The Enzalutamide-APC interaction we found in luminal A suggests a potential new application of androgen receptor antagonists in hormone receptor-positive BC [61], which may complement standard endocrine therapies. For Luminal B, pairs like Nivolumab-PMS2 and Ipilimumab-MLH1 underscore the growing role of immunotherapy in BC [62,63]. Overall, these results exemplify how our dimensionality reduction approach can facilitate the identification of actionable targets tailored to BC subtypes. Future work should focus on experimentally validating these interactions and exploring their clinical applicability in preclinical models and patient cohorts.

## Conclusion

While our primary objective was the exploration of dimensionality reduction and learning methods that have the power to provide unique markers associated with BC subtypes, we demonstrated that the reduced feature space also provides intriguing mechanistic insights. This approach offers the potential for development of diagnostic markers and novel therapeutics based on unique mechanisms. The features identified in this study could serve as a valuable input for exploring other cohorts. One limitation of this study was the size of the data. Autoencoders are unsupervised learning techniques, i.e., rather than depending on human-created labels, they essentially rely on the data itself to make inferences. Therefore, the size of the input data can play an important role for them to learn the hidden representation of the data. Our data, especially that from HER2-enriched cancer (see Table 1), was limited. Notwithstanding the data limitations, we have demonstrated that the autoencoder method yielded BC subtype-specific markers and mechanisms that have therapeutic implications. The autoencoder method can be easily extended to other large cohort cancer data towards obtaining markers and mechanisms that can aid diagnosis and therapy.

## Supporting information

S1 Fig The distribution plot of the gene expression values of the data using our identified marker sets associated with each BC subtype. The minimum (Q0), first quartile (Q1), second quartile (Q2), third quartile (Q3) and maximum (Q4) of the expression values of each subtype is as follows. TNBC: Q0 = 0, Q1 = 2.0133, Q2 = 4.1600, Q3 = 7.3323, Q4 = 40.1783; HER2-enriched: Q0 = 0, Q1 = 2.0193, Q2 = 4.1112, Q3 = 7.1811, Q4 = 41.6153; luminal A: Q0 = 0, Q1 = 2.3580, Q2 = 4.6338, Q3 = 7.9131, Q4 = 134.1335; luminal B: Q0 = 0, Q1 = 2.2493, Q2 = 4.4512, Q3 = 7.6284, Q4 = 30.8596.
(TIF)

S2 Fig The upset plot showing the overlap between the features (genes) identified in each BC subtype. (TIF)

S3 Fig ROC curves showing the performance of the random forest classifier in distinguishing breast cancer subtypes on the external validation dataset (GSE81538). (TIF)

S1 Table Performance of different machine learning algorithms in classifying breast cancer subtypes using our identified features. (PDF)

S2 Table The top four gene-drug pairs identified from DGIdb databased associated with each BC subtype. (PDF)
