# Supplementary figures and images for: An autoencoder learning method for predicting breast cancer subtypes
Source: PLoS One. 2025 Jul 23;20(7):e0327773. doi: 10.1371/journal.pone.0327773 (PMC12286384; doi:10.1371/journal.pone.0327773)

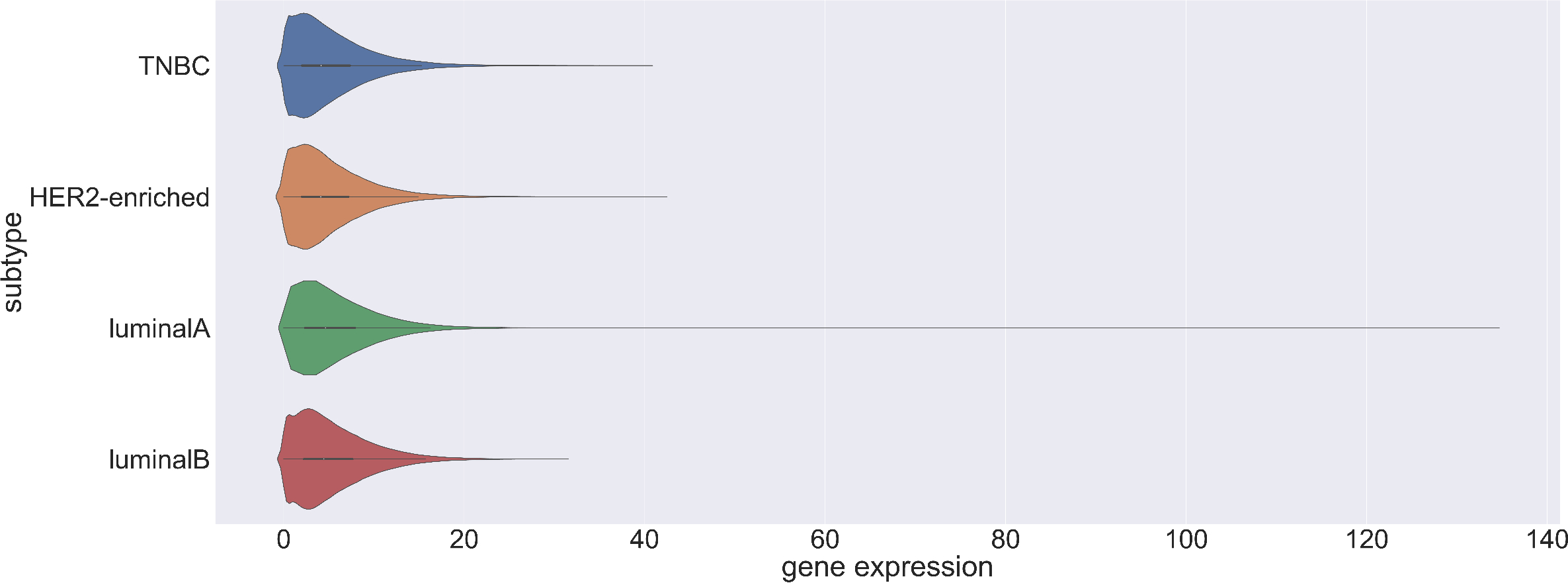

Supplement: S1 Fig — The minimum (Q0), first quartile (Q1), second quartile (Q2), third quartile (Q3) and maximum (Q4) of the expression values of each subtype is as follows. TNBC: Q0 = 0, Q1 = 2.0133, Q2 = 4.1600, Q3 = 7.3323, Q4 = 40.1783; HER2-enriched: Q0 = 0, Q1 = 2.0193, Q2 = 4.1112, Q3 = 7.1811, Q4 = 41.6153; luminal A: Q0 = 0, Q1 = 2.3580, Q2 = 4.6338, Q3 = 7.9131, Q4 = 134.1335; luminal B: Q0 = 0, Q1 = 2.2493, Q2 = 4.4512, Q3 = 7.6284, Q4 = 30.8596. (TIF) [file pone.0327773.s001.tif]

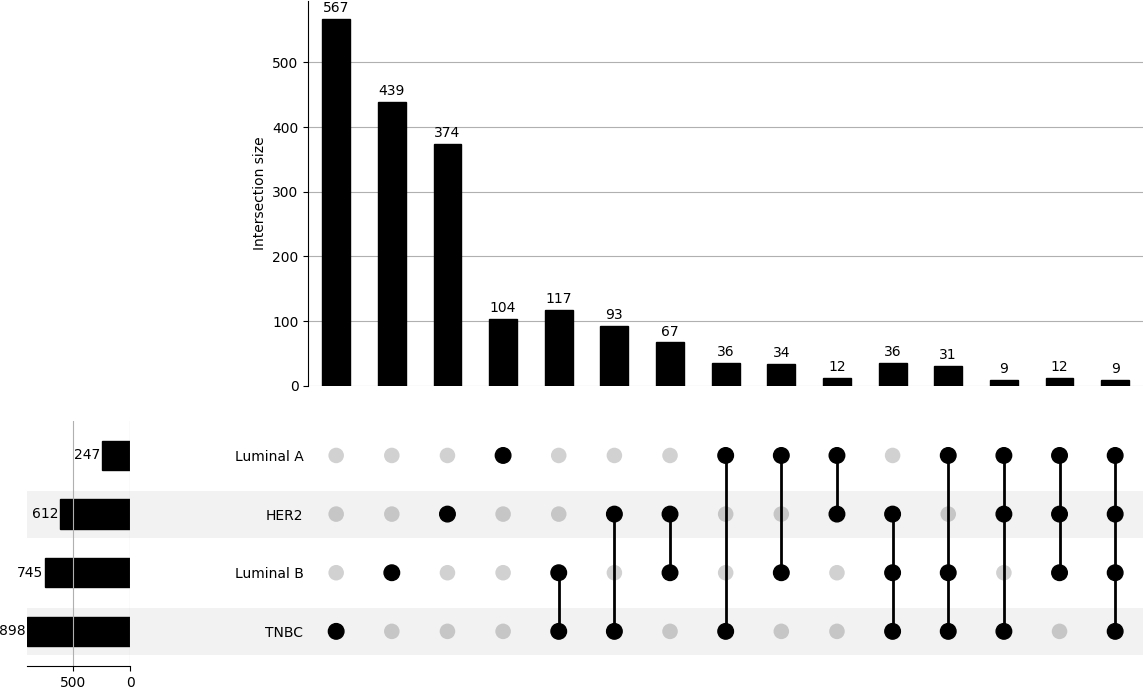

Supplement: S2 Fig — (TIF) [file pone.0327773.s002.tif]

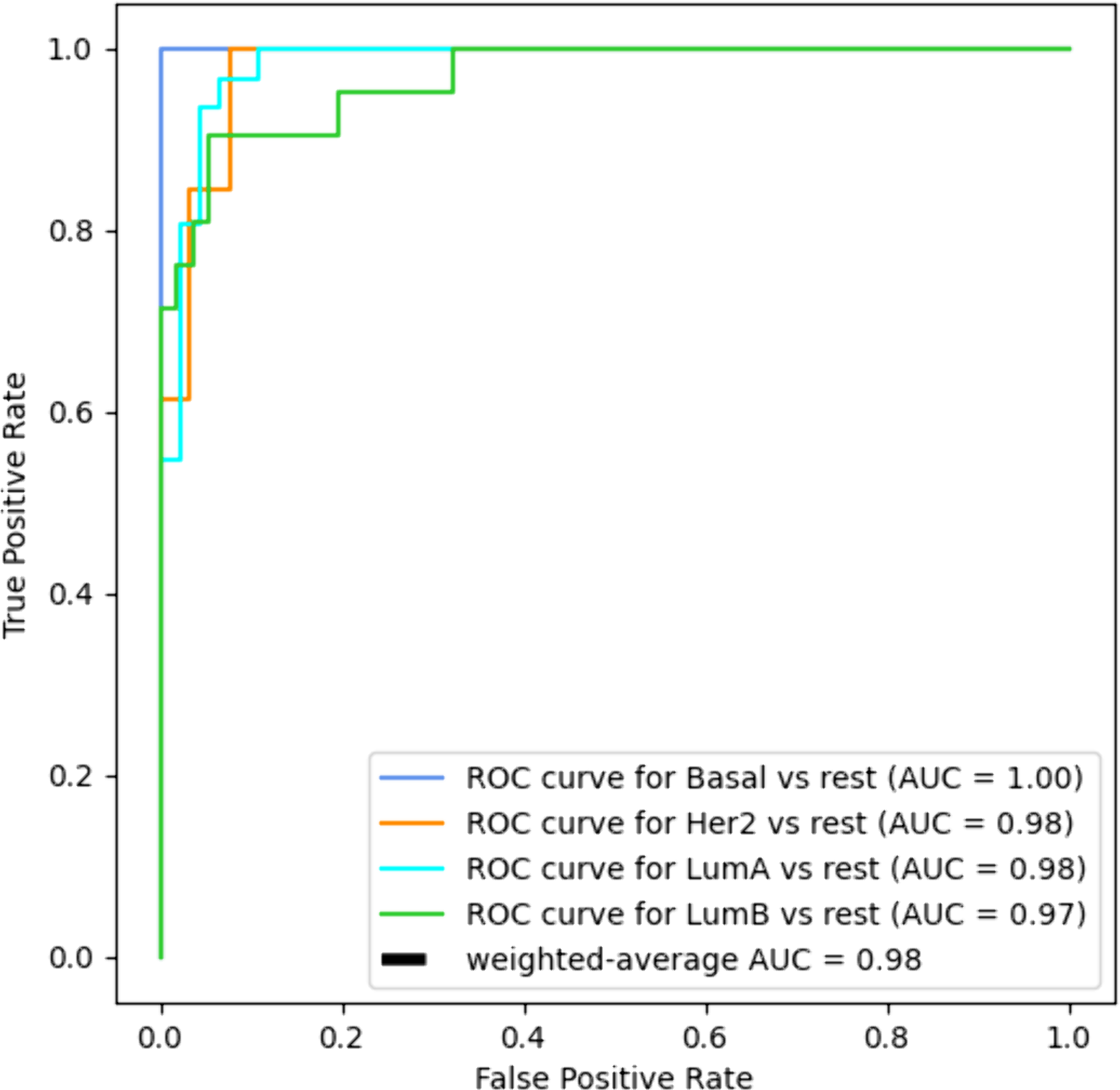

Supplement: S3 Fig — (TIF) [file pone.0327773.s003.tif]
