# Supplementary material for: An autoencoder learning method for predicting breast cancer subtypes
Source: PLoS One. 2025 Jul 23;20(7):e0327773. doi: 10.1371/journal.pone.0327773 (PMC12286384; doi:10.1371/journal.pone.0327773)
Supplement: S1 Table — (PDF) [file pone.0327773.s004.pdf]

S1 Table. Performance of different machine learning algorithms in classifying breast cancer subtypes using our identified features.

Method	Accuracy	Avg Precision	Avg Recall	Avg F1 score
Random Forest	82.38%	84%	82%	79%
Logistic Regression	82.38%	83%	82%	81%
SVM	84.45%	84%	84%	83%
