# Supplementary material for: An autoencoder learning method for predicting breast cancer subtypes
Source: PLoS One. 2025 Jul 23;20(7):e0327773. doi: 10.1371/journal.pone.0327773 (PMC12286384; doi:10.1371/journal.pone.0327773)
Supplement: S2 Table — (PDF) [file pone.0327773.s005.pdf]

S2 Table. The top four gene-drug pairs identified from DGIdb databased associated with each BC subtype.

BC Subtype	Gene	Drug
TNBC	SIN3A	Hydroxyurea
	TIRAP	Ustekinumab
	XRCC4	Docetaxel Anhydrous
	RIPK1	Resveratrol
HER2-enriched	STK11	Phenformin
	FLCN	Everolimus
	RIPK1	Resveratrol
	RAD50	Irinotecan Hydrochloride
Luminal A	PRKAB1	Metformin
	PRKAB1	Propylthiouracil
	APC	Enzalutamide
	APC	Eflornithine
Luminal B	RFC1	Pralatrexate
	SIN3A	Hydroxyurea
	MLH1	Ipilimumab
	PMS2	Nivolumab
